# Geospatial variations and predictors of low birth weight in Sub-Saharan Africa: a geospatial modeling using evidence from demographic health survey 2015–2024

**DOI:** 10.1016/j.eclinm.2025.103693

**Published:** 2025-12-18

**Authors:** Bewketu Sendek Aragie, Getaneh Awoke Yismaw, Belayneh Jejaw Abate, Ashenafi Solomon Weldeyohanis, Solomon Gedlu Nigatu

**Affiliations:** a Department of Epidemiology and Biostatistics, Institute of Public Health, College of Medicine and Health Sciences, University of Gondar, Gondar, Ethiopia; b University of Gondar Comprehensive Specialized Hospital, Gondar, Ethiopia

**Keywords:** Low birth weight, Spatial analysis, Multi-scale geographic weighted regression, Sub-Saharan Africa

## Abstract

**Background:**

Low birth weight, defined as less than 2.5 kg (5.5 lbs) at birth, remains a critical global public health challenge. It significantly increases the risk of neonatal mortality and immediate complications such as sepsis and hypothermia, along with lifelong consequences including childhood disabilities and adult-onset chronic diseases. However, there was a limited study that described the spatial distribution and predictors of low birth weight in sub-Saharan Africa. The study aimed to assess geospatial variations and predictors of low birth weight in sub-Saharan Africa.

**Methods:**

A community-based cross-sectional study design based on Demographic and Health Survey (2015–2024) data, comprising a weighted sample of 138,164 women aged 15–49 years with live births among 28 sub-Saharan African countries, was included in the study. Global Moran's I was calculated to determine overall clustering of low birth weight. Statistically significant hot spot and cold spot areas of low birth weight were determined by Getis-Ord G∗ statistics. Ordinary least squares, spatial lag, spatial error, geographically weighted regression, and multiscale geographically weighted regressions were utilized to determine predictors of low birth weight. The best-fitting models were determined by the highest R^2^ and the lowest corrected Akaike Information Criterion values. Finally, the statistically significant predictors from the final model were displayed on a map.

**Findings:**

Low birth weight was clustered (Moran's I 0.23, z-score 50.2, p-value <0.01) in the study area. Significant hotspot areas were depicted in Mauritania, Mali, Senegal, Burkina Faso, Nigeria, Gabon, Angola, Madagascar, South Africa, Lesotho, Malawi, and Ethiopia. Conversely, low-risk cold spots were observed in Uganda, Kenya, Rwanda, Burundi, Tanzania, Zambia, Zimbabwe, Cameroon, and Sierra Leone. Short birth interval, no visit to a health facility in the last year, twin birth, no media exposure, and unemployed women were significant predictors of low birth weight.

**Interpretation:**

There is spatial variation of low birth weight across different regions in sub-Saharan Africa. Significant hotspot and cold spot areas along with significant predictors were identified, which is a priority for policy makers. Targeted maternal health interventions, improved healthcare access, health education using mass media, and economic empowerment for women are recommended to reduce low birth weight.

**Funding:**

None.

**Research in context**

**Evidence before this study**

Sub-Saharan Africa carries the highest global burden of low birth weight. This study aimed to determine the spatial distribution and factors associated with low birth weight in sub-Saharan Africa (SSA). We conducted a PubMed search using MeSH terms, key phrases, and Boolean operators to identify published studies without language restrictions, using the terms “low birth weight” OR “INFANT LBW” OR “Low-Birth-Weight Infant” OR “Birth Weight” AND “geographical variation” OR “geospatial variation” OR “spatial pattern” AND “associated factors” OR “predictors” OR “covariates” OR “independent factors” AND “SSA” AND “sub-Saharan Africa” NOT “comment” NOT “case report.” Our search revealed one systematic review and numerous comprehensive studies conducted across SSA addressing the determinants of low birth weight. Nevertheless, these studies mainly used relative measures like odds ratios and relative risks, assuming uniform effects across regions and neglecting spatial variability. This limits their ability to identify the most affected communities or capture geographic variations in risk factors, which is essential for designing targeted interventions and informing local policy. Therefore, subnational low birth weight estimates are needed to address these gaps and support effective public health planning.

**Added value of this study**

To our knowledge, this is the most recent nationally representative DHS dataset used to examine the spatial distribution and determinants of low birth weight across 28 countries in SSA. Low birth weight was found to be clustered across the region, with significant hotspots identified in Mauritania, Mali, Senegal, Burkina Faso, Nigeria, Gabon, Angola, Madagascar, South Africa, Lesotho, Malawi, and Ethiopia. To address limitations of prior research, we applied an advanced geospatial technique, specifically multiscale geographically weighted regression (MGWR), to detect factors associated with low birth weight at varying spatial scales. Short birth interval, no visit to a health facility in the last year, twin birth, no media exposure, and unemployed women were significant spatial predictors of low birth weight in SSA. Notable geographic variations in these predictors were also observed across countries in SSA.

**Implications of all the available evidence**

Our findings provide new evidence that low birth weight in SSA is spatially clustered and influenced by local factors, suggesting that national health policies should prioritize hotspot regions with higher rates to enable strategic, region-specific allocation of public health resources and tailored interventions, thereby improving program efficiency and supporting evidence-based decision-making to address geographic disparities.

## Introduction

The birth weight of a child may be low as the result of being born prematurely or having restricted intrauterine growth, in comparison to other children. Low birth weight (LBW), defined as a birth weight of less than 2.5 kg (5.5 pounds) regardless of gestational age, remains a critical indicator of newborn health globally.^1^

According to United Nations International Children's Emergency Fund (UNICEF) and the World Health Organization (WHO) estimates for 2020, approximately 1 in 7 newborns, or 14.7% (about 19.8 million babies), were born with low birthweight.^2^^,^^3^ Nearly 95% of these cases occurred in developing countries, predominantly in South-central Asia and sub-Saharan Africa (SSA).^4^ In SSA, the estimated prevalence rate for LBW is around 10%,^5^ with considerable variation among individual countries such as 10% in Uganda^6^ to 17.3% in Ethiopia,^7^ and 22.9% in Mauritania.^5^

Low birth weight contributes significantly to neonatal mortality, accounting for more than one third of deaths (34%) and increasing the risk for surviving long term health and developmental issues.^1^ The vulnerability gradient between LBW and normal weight infants spans acute neonatal complications, childhood disability risks, and adult-onset chronic diseases, affecting entire health ecosystems and community well-being.^8^^,^^9^

Preterm births have immediate complications such as hypothermia and sepsis,^10^^,^^11^ as well as long-term health risks including chronic diseases, cognitive disabilities, and increased likelihood of early childhood death, reduced school performance, and behavioral issues.^12^ LBW can also result in developmental delays, sensory deficits, and heightened vulnerability to conditions like cardiovascular and chronic kidney disease.^13^ Furthermore, LBW infants typically have reduced nephron numbers (the kidney's functional filtering units), making them more vulnerable to developing chronic kidney disease later in life.^14^

Previous studies identified that chronic medical conditions, low hemoglobin levels, inadequate iron and folic acid supplementation, maternal age, maternal education, marital status, smoking, alcohol consumption, antenatal care visits, dietary counseling, and short birth intervals were significantly associated with LBW.^13^^,^^15^, ^16^, ^17^

Several policies and strategies have been implemented to reduce the incidence of low birth weight newborns by ensuring proper care during pregnancy, labor, delivery, and the postpartum period.^18^ In addition, LBW serves as a crucial indicator for assessing progress towards the global nutrition targets established by the WHO, which aims to achieve a 30% reduction in LBW by 2025.^19^ Reducing the LBW global burden is vital for lowering neonatal mortality and meeting Sustainable Development Goal (SDG) 3.2's objective of limiting neonatal deaths to ≤12 per 1000 live births across all nations.^1^^,^^20^

Despite global efforts to reduce low birth weight, it remains a significant public health concern, and the impact of various causes of LBW differs by geographic location.^5^ However, previous studies have primarily utilized relative measures such as odds ratios (ORs) and relative risks (RRs).^5^, ^6^, ^7^ While these studies successfully identify what the risk factors are, they operate on a key assumption: that a factor's effect is uniform across the entire region. They cannot answer critical questions of where the problem is most acute or for whom a specific risk factor is most influential.^21^^,^^22^ This approach inherently overlooks geographic variability and fails to effectively capture interactions between geographically influenced variables, making them ineffective for public health planning and resource allocation.^22^, ^23^, ^24^ For instance, an odds ratio cannot identify a specific cross-border hotspot where maternal health services gaps exist, nor can they detect if media exposure's effect on LBW differs between arid and coastal regions.

Why is this spatial context crucial? Because spatial analysis is fundamental for pinpointing the communities most impacted by issues, designing targeted local interventions, and allocating limited resources to the areas in greatest need.^19^^,^^24^^,^^25^ In addition, to the best of our knowledge, there was a limited study that describes the spatial distribution of low birth weight and factors contributing to it in SSA. In light of this, this study seeks to determine the spatial distribution and factors associated with low birth weight in SSA through geospatial analysis. The findings of this study will provide valuable, location-specific insights for policy recommendations and community resource mobilization.

## Methods

### Study design, data sources, and setting

A community-based cross-sectional study was conducted using nationally representative Demographic and Health Survey data between 2015 and 2024 in 28 sub-Saharan African countries.

The study utilized data obtained from the Demographic and Health Surveys (DHA), which are thorough, nationally representative studies providing insights into various health and demographic indicators in low- and middle-income countries.^26^ The DHS collects maternal, neonatal, and socioeconomic indicators essential for analyzing LBW determinants in SSA. These publicly available datasets can be accessed through the DHS Program website (www.dhsprogram.com).

The analysis includes data from 28 sub-Saharan African countries, selected based on data availability and categorized into four regions: Eastern Africa (Burundi, Ethiopia, Kenya, Malawi, Mozambique, Rwanda, Tanzania, Uganda, Zambia, Zimbabwe), Central Africa (Angola, Gabon), Western Africa (Benin, Burkina Faso, Côte d'Ivoire, Gambia, Ghana, Guinea, Liberia, Mali, Mauritania, Nigeria, Senegal, Sierra Leone, Cameroon), and Southern Africa (Madagascar, South Africa, Lesotho) (Appendix 1).

### Ethical statement

After submitting a consent note to the DHS Program, the IRB of the DHS program data archivists waived the need for additional permission, granting access to the dataset available at http://www.dhsprogram.com. The dataset remained confidential and will not be shared or transferred to any other parties. Full documentation of DHS data and ethical guidelines is publicly accessible at http://goo.gl/ny8T6X. The study adhered to the Strengthening the Reporting of Observational Studies in Epidemiology (STROBE) guidelines, specifically following the STROBE Checklist for cross-sectional studies.

### Sampling procedures and sample size

The DHS dataset utilized a two-stage stratified sampling method. In the first stage, clusters (enumeration areas) were selected with probability proportional to size, using the variable v001, which was sequentially arranged across countries to avoid overlap, totaling 17,506 clusters. In the second stage, households within clusters were chosen through equal probability systematic sampling. A total of **159,431** mothers with under-five children reported the birth weights of their children, including residents and overnight visitors, were eligible for interviews. Data weights (v005/1,000,000) were applied to ensure representativeness. The final weighted sample comprised **138,164** who had reported the birth weights of their children within the five years preceding the survey, based on DHS Kids record from 28 countries (Appendix 2).

### Outcome variables

The outcome variable in this study was the birth weight of infants, classified as either normal or low birth weight (LBW). Infants weighing 2500 g or more were classified as having a normal birth weight, while those weighing less than 2500 g are classified as low birth weight. The low birth weight (LBW) status was coded as “1” for “yes” (indicating LBW) and “0” for “no” (indicating normal weight).^1^

### Independent variables

**Socio-demographic characteristics:** women's age at delivery, place of residence, maternal education level, wealth index, mother's occupation, father's education, and marital status.

**Maternal and child-related factors:** history of taking iron supplements during pregnancy, birth interval, number of antenatal visits, history of pregnancy termination, parity, maternal age at first birth, last birth by cesarean section delivery, no visit to a health facility in the last year, and cesarean delivery.

**Child-related factors:** child's sex, twin birth, and birth order.

**Environmental-related factors:** smoking, type of cooking fuel, source of drinking water, distance to health facility, and media exposure. A complete list of all predictors, along with their measurements, can be found in Appendix 3.

### Statistical analysis

Prior to analysis, data from multiple DHS data were merged and harmonized to ensure consistency across countries and survey years. Nationally representative estimates were maintained by applying survey-specific sampling weights. Data management, including extraction, cleaning, and descriptive analysis, was performed using STATA 17, while spatial analyses were conducted in ArcGIS 10.8 and SaTScan 9.6. Missing data were observed for several variables: father's education level (4.9%), antenatal care visit during pregnancy (4.5%), father's occupation (2.3%), distance to a health facility (2.3%), cigarette smoke exposure (2.3%), mother's occupation (0.9%), last birth by cesarean section (0.3%), and visits to a health facility (0.1%). The missingness followed an arbitrary pattern, assumed to be missing at random, and was handled in accordance with DHS guidelines (Appendix 4). The African Albers Equal Area Conic Projection was used for mapping to maintain accurate area representation.^27^

### Spatial analysis

Spatial autocorrelation was assessed using global Moran's I to determine whether LBW exhibited clustering (Moran's I > 0, p < 0.05), dispersion (Moran's I < 0, p < 0.05), or randomness (p > 0.05) across SSA. While Moran's I identifies overall clustering patterns, it does not distinguish between high- and low-value clusters. To address this limitation, we used the Getis-Ord General G statistic, which specifically detects significant concentrations of high or low LBW values.

Although identifying clusters of LBW highlights spatial patterns, such analyses alone do not indicate whether specific geographic areas experience higher or lower prevalence. To address this, we conducted Hotspot Analysis using the Getis-Ord Gi∗ statistic, which enabled us to pinpoint statistically significant hotspots (areas of high LBW prevalence) and cold spots (areas of low LBW prevalence). To estimate LBW prevalence in unsampled locations, we applied ordinary Kriging interpolation, providing a continuous surface of predicted values across the study region. Furthermore, we utilized spatial scan statistics (SaTScan) with a Bernoulli model and a 50% maximum scanning window to detect and delineate high-risk clusters of LBW, enhancing the identification of areas requiring targeted interventions.

Following cluster identification, ordinary least squares (OLS) regression explored relationships between predictors and LBW rates, with model diagnostics ensuring assumptions were met, including checks for multicollinearity (VIF <7.5), normality (Jarque–Bera test), and spatial independence of residuals (Moran's I).

Recognizing spatial dependencies, spatial lag and spatial error models were employed to account for spatial autocorrelation in outcomes and residuals, respectively, improving estimate reliability. Model selection was guided by Lagrange Multiplier tests to determine the appropriate spatial regression approach (Appendix 5).

Given potential non-stationarity in variable relationships (indicated by the Koenker-Breusch-Pagan test), Geographically Weighted Regression (GWR) was used to model location-specific effects. Finally, Multiscale Geographically Weighted Regression (MGWR) extended GWR by allowing predictors to operate at different spatial scales, improving the model's accuracy in capturing spatially varying relationships. Model comparison was based on selecting the model with the lowest corrected Akaike Information Criterion (AICc) and the highest adjusted R^2^. Predictors demonstrating statistical significance (p < 0.05) in the final model were subsequently mapped to visualize their spatial distribution.

### Role of the funding source

There was no funding source for this study.

## Results

### Descriptive statistics of study variables

This study included a total weighted sample of 138,164 mothers with under-five children, drawn from the Demographic and Health Surveys (DHS) across 28 SSA countries. The majority of study participants were women aged 20–29 (50.49%) residing in rural areas (57.56%) and attaining a secondary or higher education level (40.77%) (Table 1). Detail descriptive statistics of study variables were reported in Appendix 6.

**Table 1:** Socio-demographic characteristics of the study participants in a study of spatial distribution and associated factors of low birth weight in Sub-Saharan Africa, DHS 2015–2024.

	Category	Weighted frequency	Weighted percentage
Women age in year	15_19	8816	6.38
	20_29	69,762	50.49
	30_39	49,145	35.57
	40_49	10,441	7.56
Place of residence	Urban	58,641	42.44
	Rural	79,523	57.56
Women education level	No education	34,902	25.26
	Primary	46,931	33.97
	Secondary and above	56,331	40.77
Father education level	No education	32,896	23.81
	Primary	34,100	24.68
	Secondary and above	71,168	51.51
Mother occupation	Mother do not have job	43,240	31.30
	Agriculture	36,873	26.68
	Mother had job (paid)	58,052	42.02
Father occupation	Father do not have job	10,753	7.79
	Agriculture	33,895	24.53
	Father had job (paid)	93,516	67.68
Wealth index	Poorest	48,753	35.29
	Poorer	27,412	19.84
	Middle	61,999	44.87

### Global spatial autocorrelation (Moran's I) analysis

The spatial determinants of low birth were clustered (Moran's I = 0.23, z-score is 50.2, p-value <0.01) in the study area. The finding reveals a 99% confidence level that similar values are spatially clustered more than would occur by chance (Appendix 7). In addition, the high-low clustering analysis indicates significant clustering of high values in the study area, with a z-score of 45.4 and a p-value below 0.01 (Appendix 8).

### Hot/cold spot area of low birth weight in SSA

**Fig. 1:**
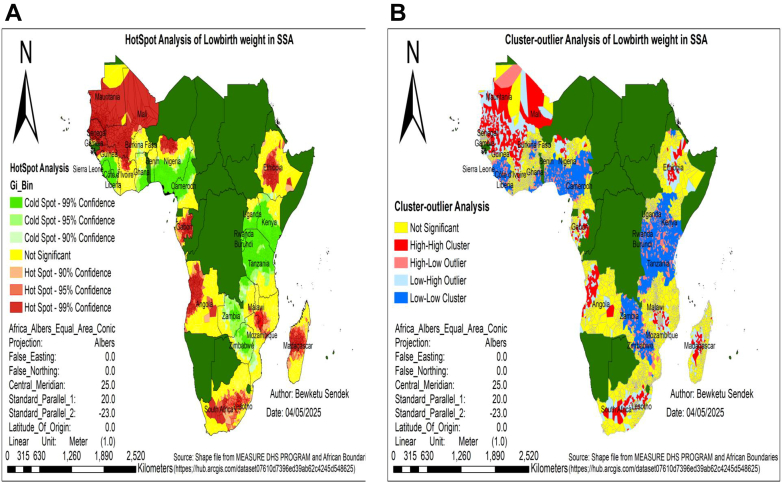
Hot spot analysis **(A)** and cluster-outlier analysis **(B)** of low birth weight in SSA, DHS 2015–2024. Notes: Red color shades in figure A and B represents countries with high low birth weight, while green color in figure A and blue color in figure B shades reveals the countries with low rate. Strength of presence is indicated by depth of color. SSA represents Sub-Saharan Africa, DHS represents demographic and health survey data.

The Getis-Ord Gi analysis identified significant spatial clustering of low birth weight, with high-risk hotspots (red) located in Mauritania, Mali, Senegal, Burkina Faso, Nigeria, Gabon, Angola, Madagascar, South Africa, Lesotho, Malawi, and Ethiopia. Conversely, low-risk cold spots (green) were predominantly observed in Uganda, Kenya, Rwanda, Burundi, Tanzania, Zambia, Zimbabwe, Cameroon, and Sierra Leone (Fig. 1A).

### Cluster-outlier analysis of low birth weight in SSA

Cluster outlier analysis identified distinct patterns of LBW across Sub-Saharan Africa. Significant high–high clusters (areas of high LBW surrounded by high LBW) were found in Mali, Mauritania, Angola, and Senegal. While low–low clusters (low LBW surrounded by low LBW) occurred in Sierra Leone, Rwanda, Burundi, and Tanzania. Additionally, low-high outliers were identified in Ethiopia, Madagascar, Nigeria, Gabon, and South Africa, where low birth weight rates were surrounded by high rates, while high-low outliers were found in Uganda, Kenya, Zambia, Zimbabwe, Cameroon, Côte d'Ivoire, and Burkina Faso, where high rates were surrounded by low rates (Fig. 1B).

### Spatial interpolation analysis of low birth weight

**Fig. 2:**
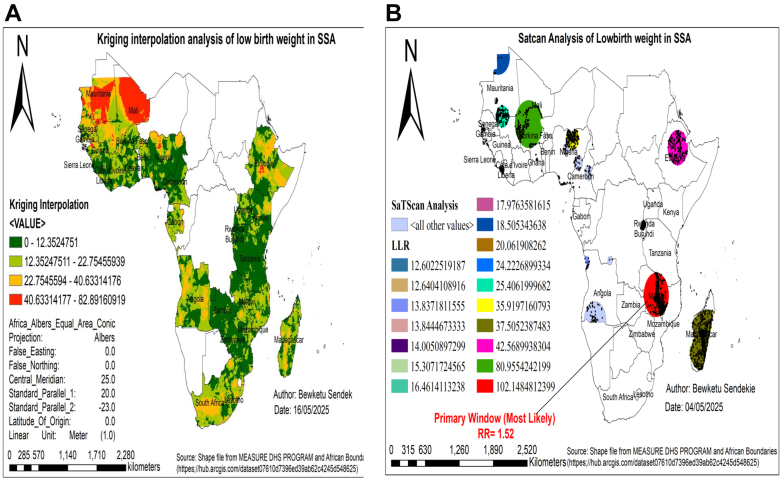
Spatial interpolation **(A)** and satscan analysis **(B)** analysis of low birth weight in SSA, DHS 2015–2024. Notes: SSA represents Sub-Saharan Africa, DHS represents demographic and health survey data. C.I. denotes confidence interval, and LLR denotes log likelihood ratio.

The ordinary kriging spatial interpolation method indicated that areas with higher predicted rates of low birth weight are shown in red, while lower rates are represented in light blue. This analysis identified Mali, Mauritania, Senegal, Nigeria, Ethiopia, and South Africa as the highest-risk areas for low birth weight, whereas low-risk areas were found in Tanzania, Rwanda, Burundi, Zambia, Zimbabwe, Cameroon, Kenya, and Sierra Leone (Fig. 2A).

### SatScan analysis of low birth weight in SSA

A total of 2982 significant enumeration areas were detected using Kulldorff's spatial scan statistic. Out of those, 886 were in the primary cluster window, and 2096 were secondary clusters. The primary cluster was located in Malawi, centered at 13.021490 S, 33.466697 E, with a radius of 389.87 km and a log-likelihood ratio (LLR) of 102.1 at p < 0.01. Neonates within this area had a 1.52 times higher risk of low birth weight compared to those outside the spatial window (Fig. 2B). The analysis also revealed 16 secondary cluster locations, as detailed in Appendix 9.

### Geographic factors influencing low birth weight in sub-Saharan Africa

Before conducting OLS regression, exploratory analysis identified the best candidate variables with at least 75% significance (Appendix 10). From these, five key predictors were selected: short birth interval, twin birth, lack of media exposure, no health facility visits in the past year, and maternal unemployment. These variables were selected based on significant coefficients, an adjusted R^2^ above 50%, a non-significant Jarque–Bera test (0.22), and low multicollinearity (condition number 4.27). The OLS model explained 50.5% of the variance in low birth weight across SSA districts. However, significant spatial clustering of low birth weight (Moran's I, p < 0.01) violated the OLS assumption of spatial independence, prompting the use of spatial dependence models. Both Lagrange Multiplier tests showed strong significance (LM-Lag = 908.9, p < 0.01; LM-Error = 872.6, p < 0.01), confirming OLS's inability to address spatial autocorrelation (Appendix 11). These results demonstrate that spatial regression approaches (SLM/SEM) are required to properly account for the spatial processes underlying low birth weight patterns in SSA (Appendix 12). However, its assumption of spatial stationarity limits its ability to capture localized variations, necessitating further analysis using geographically weighted regression (GWR) and multiscale GWR (MGWR) to explore spatially heterogeneous relationships.

Recognizing that global models fail to capture localized relationships, we employed geographically weighted regression (GWR) and multiscale GWR (MGWR). Among all models, the multiscale geographically weighted regression (MGWR) model outperformed other models with the highest explanatory power (adjusted R^2^ = 67.7%) and lowest AICc (29092.9), demonstrating its superiority in capturing localized spatial variations in low birth weight across Sub-Saharan Africa (Appendix 13).

**Fig. 3:**
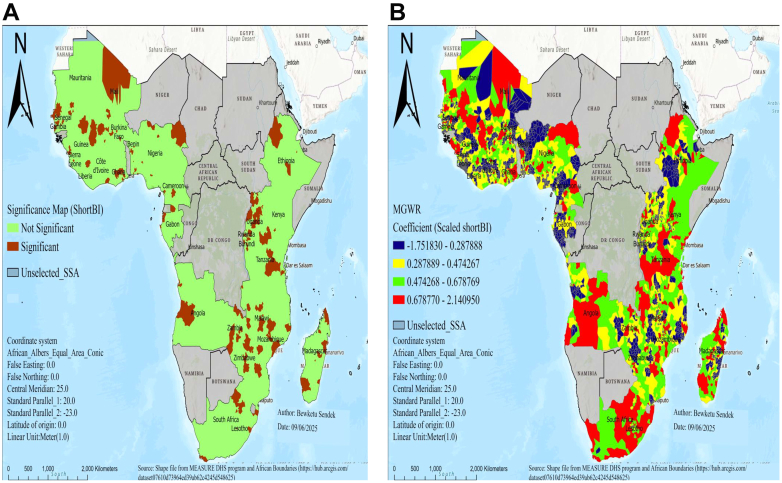
MGWR result of significant areas **(A)** and coefficients of short birth interval **(B)** for estimating low birth weight in SSA, DHS 2015–2024. Notes: Coefficients estimates and significance maps are obtained from MGWR regression. Short BI denotes short birth interval, DHS denotes demographic and health survey data, and MGWR represents multi-scale geographic weighted regression.

**Fig. 4:**
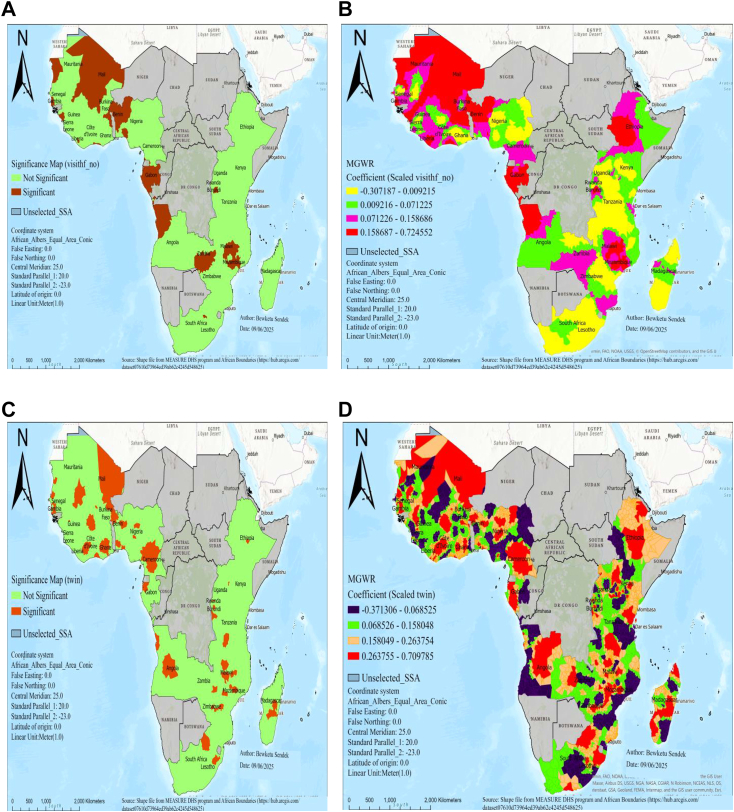
MGWR result of significant areas and coefficients no visit health facility in the last year (A and B) and twin births (C and D) for estimating low birth weight in SSA, DHS 2015–2024. Notes: Coefficient estimates and significance maps are obtained from MGWR regression. Visithf_no denotes no visit health facility in the last year, Twin denotes twin births, DHS denotes demographic and health survey data and MGWR represents multi-scale geographic weighted regression.

**Fig. 5:**
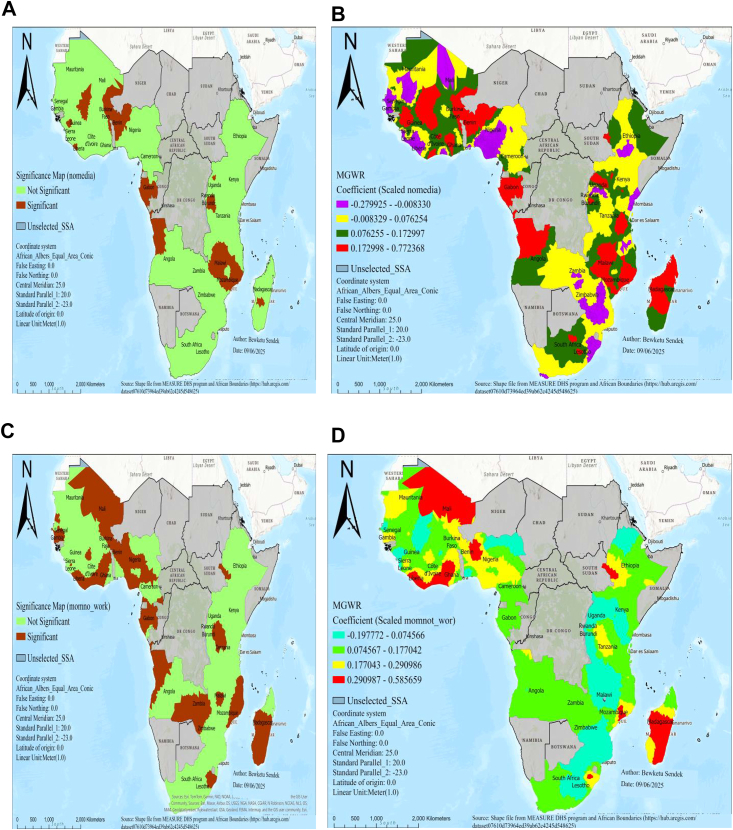
MGWR result of significant areas and coefficients of nomedia exposure (A and B) and women who have not job (C and D) for estimating low birth weight in SSA, DHS 2015–2024. Notes: Coefficient estimates and significance maps are obtained from MGWR regression. nomedia denotes no media exposure, Momnot_wor denotes women who donot have job, DHS denotes demographic and health survey data, and MGWR represents multi-scale geographic weighted regression.

Regarding spatial predictors of low birth weight in SSA, MGWR output produced predicted low birth weight maps of geographic areas where short birth interval, no visit to a health facility in the last year, twin birth, no media exposure, and unemployed women were significant predictors of low birth weight in SSA, as shown in Fig. 3, Fig. 4, Fig. 5.

There was a statistically significant association between low birth weight and short birth interval in areas of Mali, Senegal, Nigeria, Ethiopia, Gambia, Ghana, Burkina Faso, Angola, Uganda, Rwanda, Burundi, Tanzania, Malawi, Zambia, Mozambique, Zimbabwe, and Madagascar (Fig. 3A). In significant areas, as the percentage of short birth intervals increased, the occurrence of LBW also increased in areas of Mali, Nigeria, Ethiopia, Tanzania, Angola, Zimbabwe, South Africa, Lesotho, Ghana, Zambia, Senegal, Burkina Faso, and Mozambique (Fig. 3B).

There was a statistically significant association between women who have not visited a health facility and low birth weight in regions of Mali, Benin, Gambia, Gabon, Zambia, Liberia, Mauritania, Sierra Leone, Malawi, Mozambique, and Burundi (Fig. 4A). In significant areas, as the percentage of women who have not visited a health facility in the last year increased, the occurrence of low birth weight also increased in regions of Mali, Mauritania, Benin, Liberia, Gambia, Gabon, Mozambique, Ethiopia, and Malawi (Fig. 4B).

There was a statistically significant association between twin birth and low birth weight in areas of Mali, Senegal, Gambia, Burkina Faso, Benin, Ghana, Cote d’Ivoire, Nigeria, Cameroon, Gabon, Angola, Malawi, Mozambique, Zimbabwe, Burundi, and Madagascar (Fig. 4C). In significant areas, as the percentage of twin births increased, the occurrence of low birth weight also increased in areas of Mali, Nigeria, Cameroon, Ethiopia, Gabon, Angola, Malawi, Mozambique, Burundi, Zimbabwe, and Madagascar (Fig. 4D).

There was a statistically significant association between no media exposure and low birth weight in areas of Burkina Faso, Benin, Guinea, Liberia, Gabon, Angola, Malawi, Mozambique, Rwanda, Burundi, and Madagascar (Fig. 5A). In significant areas, as the percentage of no media exposure increased, the occurrence of low birth weight also increased in areas of Burkina Faso, Benin, Guinea, Liberia, Gabon, Angola, Malawi, Mozambique, and Madagascar (Fig. 5B).

There was a statistically significant association between unemployed women and low birth weight in areas of Mali, Burkina Faso, Benin, Liberia, Senegal, Gambia, Liberia, Nigeria, Gabon, Angola, Zambia, Malawi, Mozambique, Lesotho, Madagascar, Ethiopia, and Tanzania (Fig. 5C). In significant areas, a 10% increase in women who don't have jobs results in a 2.9–5.8% rise in low birth weight in areas of Mali, Ghana, Liberia, Benin, Mozambique, and Madagascar (Fig. 5D).

## Discussion

Spatial analysis plays a crucial role in identifying high-risk areas, enabling more effective intervention targeting and the development of tailored local strategies.^27^ In this study, an attempt has been made to assess the spatial variations and predictors of low birth weight in SSA using DHS 2015–2024 data.

The spatial variation of low birth weight was clustered across regions in SSA, with a global Moran's index value of 0.23 (p < 0.01). This showed that there was a significant clustering of low birth weight in SSA across the region. The spatial analysis identified hotspot areas of low birth weight in Mauritania, Mali, Senegal, Burkina Faso, Nigeria, Gabon, Angola, Madagascar, South Africa, Lesotho, Malawi, and Ethiopia. Conversely, low-risk cold spots were detected in Uganda, Kenya, Rwanda, Burundi, Tanzania, Zambia, Zimbabwe, Cameroon, and Sierra Leone. The discrepancies are linked to agroecological disparities and climate-driven food insecurity.^5^^,^^28^^,^^29^ For instance, hotspot countries experience rain-fed agriculture, prolonged dry seasons, and recurrent droughts, which exacerbate maternal undernutrition and limit access to nutrient-rich diets during pregnancy.^5^^,^^28^ These places also face higher malaria exposure and heat stress, both of which impair fetal growth.^28^^,^^29^ Furthermore, high poverty rates, limited maternal healthcare infrastructure, and lower female education levels contribute to poor maternal nutrition and inadequate prenatal care, leading to maternal malnutrition and micronutrient deficiencies.^5^ Low-risk cold spots for low birth weight likely originate from the combined effect of improved maternal education, improved access to antenatal care, improved maternal nutrition, successful public health interventions, and favorable socioeconomic conditions that jointly result in healthier birth outcomes in these countries.^15^^,^^30^

In the MGWR model, short birth interval not visiting a health facility in the last 1 year, twin birth, no media exposure, and mom not having jobs were statistically significant predictors of LBW.

Short birth interval had a positive association with LBW in most of SSA. As the percentage of short birth intervals increased, the occurrence of LBW also increased in areas of Mali, Nigeria, Ethiopia, Tanzania, Angola, Zimbabwe, South Africa, Lesotho, Ghana, Zambia, Senegal, Burkina Faso, and Mozambique. This might be primarily due to maternal nutritional depletion and insufficient recovery time between pregnancies.^31^^,^^32^ When pregnancies occur in rapid succession, the mother's body may not have enough time to restore essential nutrients and energy reserves needed to support fetal growth, increasing the risk of delivering a low birth weight baby.^31^ In addition, limited access to quality antenatal and maternal healthcare services, especially in underserved regions,^5^ and localized factors like severe maternal anemia, poverty, and lower maternal education might contribute to the above discrepancies.^29^ Furthermore, variations in birth spacing practices, influenced by cultural norms and access to family planning services, differ geographically, impacting the frequency of short birth intervals and associated LBW risks.^5^^,^^15^ Extending the interval between pregnancies through effective family planning and maternal care can reduce the incidence of low birth weight.

As the percentage of women who have not visited a health facility in the last year increased, the occurrence of low birth weight also increased in areas of Mali, Mauritania, Benin, Gambia, Liberia, Gabon, Mozambique, Ethiopia, and Malawi, which also displayed significant hotspots of low birth weight. This might be due to interconnected socioeconomic, climatic, cultural, and systemic barriers.^28^^,^^33^ For example, high temperatures and low agricultural production in Mali limit women from accessing health care and result in LBW.^28^ In addition, many women in those identified high-risk regions face significant financial barriers that make healthcare unaffordable, leading to adverse birth outcomes.^34^ Without access to health services, they miss critical interventions like malaria prevention, HIV testing and treatment, and nutritional support, which help prevent low birth weight.^35^

As the percentage of twin births increased, the occurrence of low birth weight also increased in regions including Mali, Nigeria, Cameroon, Ethiopia, Gabon, Angola, Malawi, Mozambique, Burundi, Zimbabwe, and Madagascar. This can be attributed to multiple interconnected factors. Sub-Saharan Africa's exceptionally high twinning rates, averaging 17 per 1000 births compared to the global average of 11 per 1000, create a larger population at risk. This elevated twinning frequency stems primarily from genetic predispositions, particularly in West and Central Africa's “Twin Belt”.^36^ The biological nature of twin pregnancies inherently increases LBW risks through several mechanisms. Twins frequently experience preterm delivery, resulting in less gestational time for proper growth and development.^37^ In addition, the shared uterine environment is usually accompanied by competition for oxygen and nutrients and in most instances leads to intrauterine growth restriction. These biological challenges are compounded by higher rates of pregnancy complications like preeclampsia and placental insufficiency, which impair fetal growth and increase the likelihood of low birth weight.^37^^,^^38^ Carrying multiples places greater nutritional and physiological demands on the mother, which can be challenging, especially in settings with maternal undernutrition or limited prenatal care, further increasing LBW risk.^38^

Similarly, as the percentage of no media exposure increased, low birth weight also increased in countries including Burkina Faso, Benin, Guinea, Liberia, Gabon, Angola, Malawi, Mozambique, and Madagascar. This might be linked to information access and health education disparities.^5^ For instance, poor infrastructure (such as limited electricity and weak telecommunications networks), geographic barriers like difficult terrain and remote rural locations, low literacy rates, socioeconomic challenges, and under-resourced health systems all restrict access to mass media and health information.^5^^,^^39^ Limited access to vital health information and education reduces maternal awareness about proper nutrition, antenatal care, and pregnancy health practices, thereby increasing the risk of adverse birth outcomes like low birth weight.^5^

Likewise, as the percentage of women who don't have jobs increased, the occurrence of low birth weight also increased in areas of Mali, Ghana, Liberia, Benin, Mozambique, and Madagascar. This might be due to the socioeconomic disadvantages linked to lack of employment. Unemployed women also suffer from economic dependence, limiting purchasing capacity to purchase nutritious foods and obtain quality antenatal care, which are both crucial to allow normal fetal development.^40^ In addition, unemployment is more common in rural and geographically isolated areas where healthcare services and health education are less accessible, exacerbating risks for LBW.^41^ The lack of income also increases psychosocial stress and food insecurity, which negatively affect pregnancy outcomes.^40^^,^^42^ Thus, geographic isolation combined with socioeconomic vulnerability tied to women's unemployment contributes significantly to higher LBW rates in these regions.^5^^,^^43^

This study has several strengths; the study utilizes nationally representative DHS data of large sample size, which enables the robust estimation of the spatial distribution of LBW in SSA. These results are critical in planning interventions to promote Sustainable Development Goal (SDG 3) 2030 targets. In addition, the application of sophisticated geospatial regression models allows the determination of region-specific predictors of LBW, making policy planning more accurate. However, some limitations must be acknowledged. First, the data were collected over different time periods across countries. While this variation could potentially introduce bias due to shifting trends in LBW risk factors, the large number of countries and the substantial sample size help to mitigate this concern by providing a broad and stable cross-sectional overview of the region. Second, the geographical locations of clusters were displaced for privacy reasons (up to 2 km for urban areas and 5 km for rural areas). This displacement could affect the precision of our spatial analysis. Nevertheless, the aggregate patterns at each enumeration areas remain valid and are sufficient for informing public health planning. Finally, the cross-sectional nature of the data restricts the ability to establish causal relationships. The identified associations should be interpreted as spatial correlational rather than causal.

In conclusion, low birth weight was geographically varied across regions in SSA. Hotspot regions for low birth weight were detected in Mauritania, Mali, Senegal, Burkina Faso, Nigeria, Gabon, Angola, Madagascar, South Africa, Lesotho, Malawi, and Ethiopia. Short birth interval, no visit to a health facility in the last year, twin birth, no media exposure, and unemployed women were significant predictors of LBW. Targeted maternal health interventions, improved health care access, media-based health education, economic empowerment for unemployed women, and specialized care for twin pregnancies are urgently needed to mitigate the significant spatial clustering of LBW observed across high-risk SSA countries. We suggest that public health programs and various stakeholders collaborate with other sectors, concentrating their efforts on the identified hotspot regions of low birth weight.

## Contributors

B.S.A.: conceptualization, data duration, formal analysis, methodology, software, and writing–original draft. B.J.A., G.A.: writing–review and editing, supervision. A.S.W., S.G.N.,: visualization. B.J.A., G.A., and S.G.N.: accessed and verified the data. B.J.A. and B.S.A. made the decision to submit the manuscript, with B.S.A. serving as the guarantor for the work. All authors read and approved the final version of the manuscript.

## Data sharing statement

The study utilized data from the Demographic and Health Surveys (DHS), with details on the dataset and its contents accessible at https://dhsprogram.com/data/available-datasets.cfm.

## Editor note

The Lancet Group takes a neutral position with respect to territorial claims in published maps and institutional affiliations.

## Declaration of interests

All authors declare no competing interests.
