# Supplementary material for: Geospatial variations and predictors of low birth weight in Sub-Saharan Africa: a geospatial modeling using evidence from demographic health survey 2015–2024
Source: eClinicalMedicine. 2025 Dec 18;91:103693. doi: 10.1016/j.eclinm.2025.103693 (PMC12774685; doi:10.1016/j.eclinm.2025.103693)
Supplement: Supplementary Materials [file mmc1.docx]

Appendix 1: Study area map


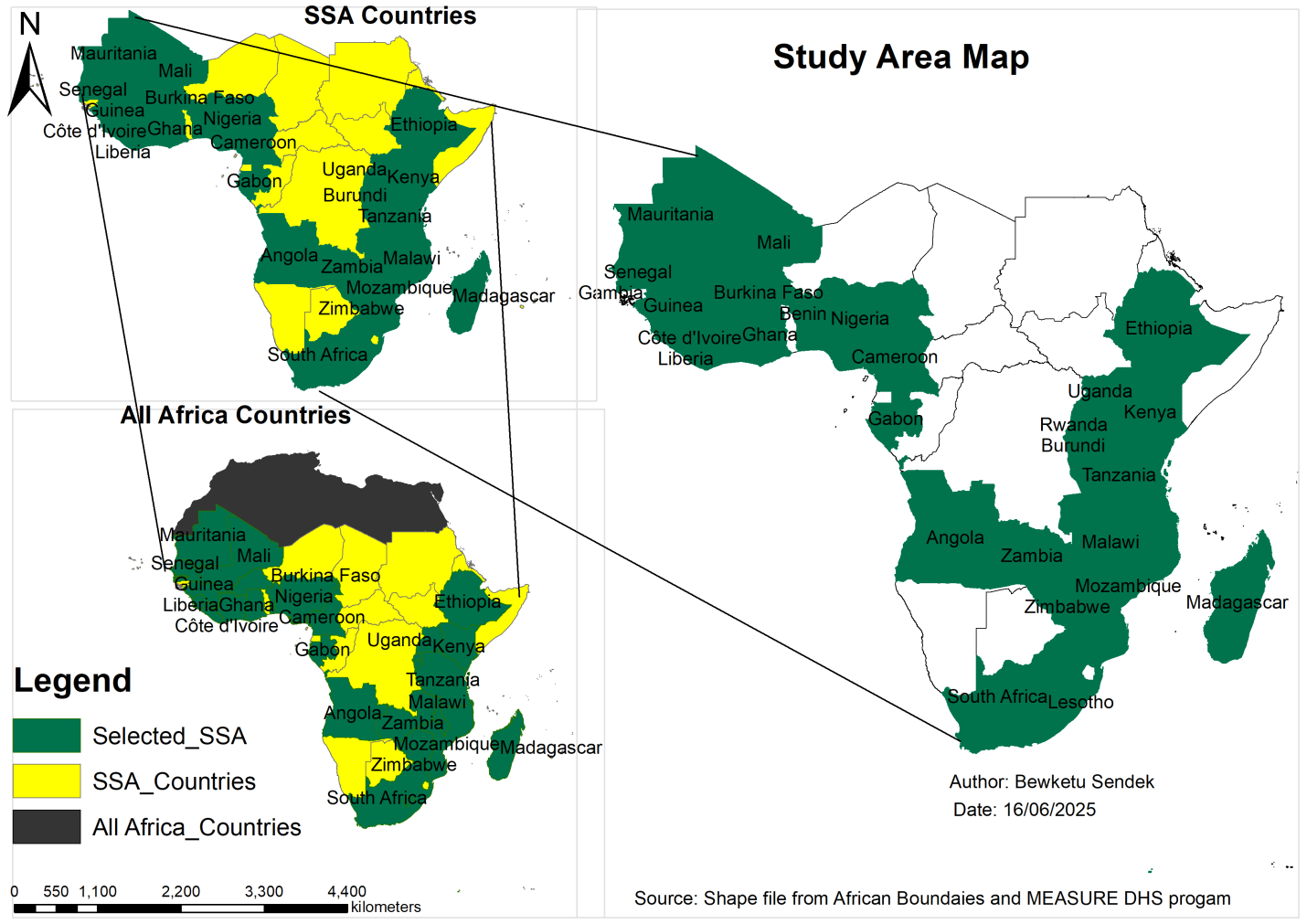


Figure 1: Study area map for determining spatial variation of low birth weight in SSA: DHS 2015-2024.

*Notes: The results are obtained from ArcGIS software. The black color shade indicates all African countries, which are out of SSA countries; the yellow color shade denotes all sub-Saharan African countries; and the green color denotes the 28 countries selected for study. DHS denotes Demographic and Health Survey, and SSA denotes sub-Saharan African countries.*

##

## Appendix 2: Sampling procedure for the study of spatial variation and determinants of low birth weight in SSA, DHS 2015-2024.

Sub Saharan Africa Countries (SSA)

N=46

SSA that have DHS data sets (n=40)

SSA countries that lack DHS datasets (n=6).

(n=40)

Selected SSA countries (n=28)

Sub-Saharan African countries prior to 2015 (N=12).

Enumeration areas (EAs) for Selected SSA countries (n=17,506)

Mothers with under-five children (n= **313,277**)

Mothers with under-five children reported the birth weights of their children.

(n= **159,431**)

Final weighted sample size for the study **(n=138,164)**

Excluded due to macrosomia (n=21,267).

## Appendix 3: Measurement of predictors

| Socio demographic characteristics of participants | |
| --- | --- |
| Predictors | Category |
| Women age | 15_19 |
|  | 20_29 |
|  | 30_39 |
|  | 40_49 |
| Place of residence | Urban |
|  | Rural |
| Women education level | No education |
|  | Primary |
|  | Secondary and above |
| Father education level | No education |
|  | Primary |
|  | Secondary and above |
| Mother occupation | Mother do not have job |
|  | Agriculture |
|  | Mother had job(paid) |
| Father occupation | Father do not have job |
|  | Agriculture |
|  | Father had job(paid) |
| Wealth index | Poorest |
|  | Poorer |
|  | Middle |
| Maternal and obstetrics related factors | |
| Maternal age at first birth | Less/equal 18 year |
|  | Above 18 year |
| During pregnancy, given or bought iron tablets/syrup | Yes |
|  | No |
| Preceding birth interval | Less than 24 month |
|  | 24 and above |
| ANC visit during pregnancy | Not at all |
|  | 1_3 |
|  | Four and above |
| History of terminated pregnancy | Yes |
|  | No |
| Total children ever born | One |
|  | 2_3 |
|  | 4 and above |
| Last birth a cesarean section | Yes |
|  | No |
| Women access to decision making | Yes |
|  | No |
| Desire for more children | Active desire |
|  | No active desire |
| Visit health facility in the last 12 months | Yes |
|  | No |
| Child related factors | |
| Sex of child | Male |
|  | Female |
| Twin birth | Yes |
|  | No |
| Birth order | 1 |
|  | 2_3 |
|  | 4 and above |
| Environmental related factors | |
| Covered by health insurance | Yes |
|  | No |
| Distance from health facility | Big problem |
|  | No problem |
| Media exposure | Yes |
|  | No |
| Source of drinking water | Improved |
|  | Unimproved |
| Cigarette smoke | Yes |
|  | No |
| Type of cooking fuel | Solid fuel |
|  | Clean fuel |

Appendix 4: Missing data management

1. **Missing Pattern**

Missing data mechanisms can be classified as one of the following;

- MCAR: Missing completely at random,
- MAR: Missing at random, or
- MNAR: Missing not at random.

**Missing completely at random**

- The probability that data is missing is unrelated to both the observed data and the unobserved data. In other words, the missingness is a completely random subset of the entire dataset.
- **Example:** A paper survey form is damaged in the mail, making some answers unreadable. The damage is unrelated to the content of the survey responses.

**Missing at random**

- The probability that data is missing is related to other observed variables in the dataset but not to the unobserved value itself.
- The term "at random" is slightly misleading. It does not mean the missingness is random. It means that once we account for the observed data, the reason for the missingness is random. The missingness can be explained by what we can see.
- **Example:** older women might be more likely to have missing data on the variable "age at first birth" because it was longer ago and they don't remember. The missingness is related to the observed variable "current age".

**Missing Not at Random**

- The probability that data is missing is directly related to the unobserved value itself, even after controlling for other observed variables. This is also known as non-ignorable missingness.
- This is the most difficult type of missing data to handle because the reason for the missingness is directly tied to the value that is missing. The "missingness" itself carries information about the missing value.
- **Example:** In wealth surveys, very wealthy individuals may be more likely to refuse to answer questions about their income or assets.

Out of the 25 predictors, 8 variables had missing data. To assess the extent of missingness, the ‘mdesc’ package in Stata was utilized. The missing data patterns were explored descriptively by examining the frequency distributions of the predictors, although this approach was somewhat arbitrary **(Table 1).** The types of missingness were further investigated by conducting regression analyses to evaluate whether the missingness of a variable was associated with other explanatory variables. The missing data were found to be missing at random (MAR) and were handled in accordance with guide to DHS statistics **(Table 2).**

**Table 1:** Missing-value patterns (1 means complete).

| Percent | Pattern | | | | | | | |
| --- | --- | --- | --- | --- | --- | --- | --- | --- |
|  | 1 | 2 | 3 | 4 | 5 | 6 | 7 | 8 |
| 63% | 1 | 1 | 1 | 1 | 1 | 1 | 1 | 1 |
| 19 | 1 | 1 | 1 | 1 | 1 | 1 | 1 | 0 |
| 12 | 1 | 1 | 1 | 1 | 1 | 0 | 0 | 1 |
| 2 | 1 | 1 | 1 | 1 | 1 | 0 | 0 | 0 |
| <1 | 1 | 1 | 1 | 0 | 0 | 0 | 0 | 1 |
| <1 | 1 | 1 | 1 | 0 | 0 | 1 | 1 | 1 |
| <1 | 1 | 1 | 0 | 1 | 1 | 1 | 1 | 1 |
| <1 | 1 | 1 | 1 | 0 | 0 | 1 | 1 | 0 |
| <1 | 1 | 1 | 1 | 1 | 1 | 1 | 0 | 1 |
| <1 | 1 | 1 | 0 | 1 | 1 | 1 | 1 | 0 |
| <1 | 1 | 1 | 1 | 0 | 0 | 0 | 0 | 0 |
| <1 | 1 | 0 | 1 | 1 | 1 | 1 | 1 | 1 |
| <1 | 0 | 1 | 1 | 1 | 1 | 0 | 0 | 1 |
| <1 | 1 | 1 | 1 | 1 | 1 | 0 | 1 | 1 |
| <1 | 1 | 1 | 1 | 0 | 0 | 0 | 1 | 1 |
| <1 | 1 | 1 | 0 | 1 | 1 | 0 | 0 | 1 |
| <1 | 1 | 1 | 1 | 1 | 1 | 1 | 0 | 0 |
| <1 | 1 | 0 | 1 | 1 | 1 | 1 | 1 | 0 |
| <1 | 1 | 1 | 1 | 1 | 1 | 0 | 1 | 0 |
| <1 | 1 | 0 | 1 | 1 | 1 | 0 | 0 | 1 |
| <1 | 0 | 1 | 1 | 1 | 1 | 0 | 0 | 0 |
| <1 | 1 | 1 | 0 | 1 | 1 | 0 | 0 | 0 |
| <1 | 1 | 1 | 1 | 0 | 0 | 0 | 1 | 0 |
| <1 | 1 | 1 | 1 | 0 | 0 | 1 | 0 | 1 |
| <1 | 1 | 0 | 0 | 1 | 1 | 1 | 1 | 1 |
| <1 | 1 | 0 | 0 | 1 | 1 | 1 | 1 | 0 |
| <1 | 1 | 1 | 0 | 1 | 1 | 0 | 1 | 0 |
| <1 | 1 | 0 | 1 | 1 | 1 | 0 | 0 | 0 |
| <1 | 1 | 1 | 0 | 1 | 1 | 0 | 1 | 1 |
| <1 | 1 | 1 | 1 | 0 | 0 | 1 | 0 | 0 |
| 100% |  |  |  |  |  |  |  |  |

Variables are (1) visited health facility last 12 months, (2) last birth a caesarean section (3) respondent's occupation (4) smokes cigarettes (5) distance to health facility (6) husband/partner's occupation (7) husband/partner's educational attainment (8) number of antenatal visits during pregnancy

**Table 2:** Missing data management according to guide to DHS statistics (<https://www.dhsprogram.com/pubs/pdf/DHSG1/Guide_to_DHS_Statistics_DHS-7_v2.pdf>)

| Variables | Missing | Total | Percent missing | Missing data management according to DHS guideline |
| --- | --- | --- | --- | --- |
| Women age in year | 0 | 138,164 | 0.00 | Don’t have missing value |
| Place of residence | 0 | 138,164 | 0.00 | Don’t have missing value |
| Women education level | 0 | 138,164 | 0.00 | Don’t have missing value |
| Father education level | 6525 | 138,164 | 4.9 | Included as no formal education |
| Mother occupation | 1,257 | 138,164 | 0.9 | Missing data and “don’t know” responses are reported as no occupation |
| Father occupation | 3241 | 138,164 | 2.3 | Missing data and “don’t know” responses are reported as no occupation |
| Wealth index | 0 | 138,164 | 0.00 | Don’t have missing value |
| Maternal age at first birth | 0 | 138,164 | 0.00 | Don’t have missing value |
| Preceding birth interval | 0 | 138,164 | 0.00 | Don’t have missing value |
| ANC visit during pregnancy | 6280 | 138,164 | 4.5 | Recoded into a category for no antenatal care |
| History of terminated pregnancy | 0 | 138,164 | 0.00 | Don’t have missing value |
| Total children ever born | 0 | 138,164 | 0.00 | Don’t have missing value |
| Last birth by cesarean section | 364 | 138,164 | 0.3 | Excluded from numerator of percentage delivered by Caesarean section (assumed not Caesarean section). |
| Women access to decision making | 0 | 138,164 | 0.00 | Don’t have missing value |
| Visit health facility in the last 12 months | 139 | 138,164 | 0.1 | Included as no visit |
| Desire for more children | 0 | 138,164 | 0.00 | Don’t have missing value |
| Sex of child | 0 | 138,164 | 0.00 | Don’t have missing value |
| Twin birth | 0 | 138,164 | 0.00 | Don’t have missing value |
| Birth order | 0 | 138,164 | 0.00 | Don’t have missing value |
| Covered by health insurance | 0 | 138,164 | 0.00 | Don’t have missing value |
| Distance from health facility | 3,185 | 138,164 | 2.3 | Included as not a big problem |
| Media exposure | 0 | 138,164 | 0.00 | Don’t have missing value |
| Source of drinking water | 0 | 138,164 | 0.00 | Don’t have missing value |
| Cigarette smoke | 3,185 | 138,164 | 2.3 | Included as no cigarette smoke |
| Type of cooking fuel | 0 | 138,164 | 0.00 | Don’t have missing value |

Appendix 5: Decision tree of global regression model for determining low birth weight in SSA: DHS 2015-2024.


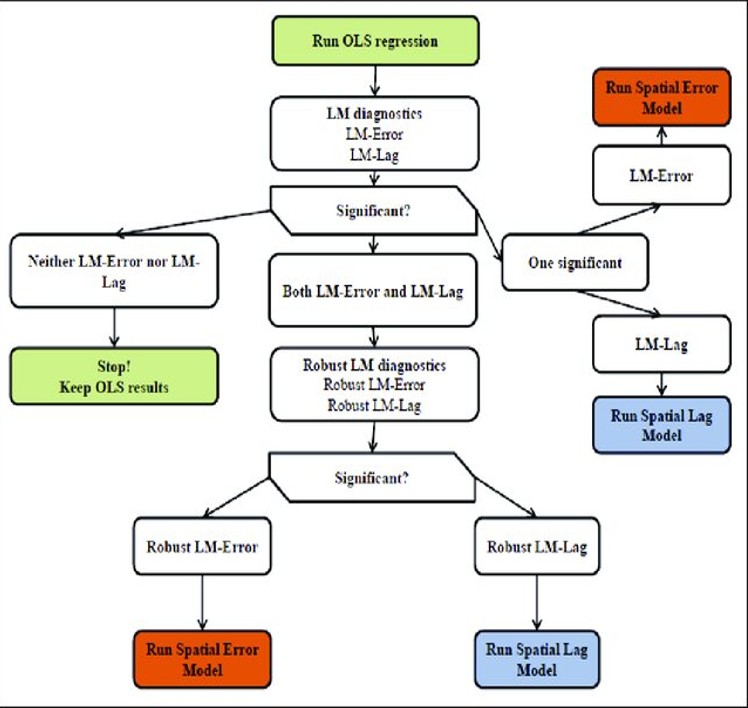


**Appendix 6: Descriptive statistics of study variables**

1. **Maternal and obstetric factors**

The majority of mothers had their first birth after age 18 (58.89%), received iron supplementation during pregnancy (91.4%), and attended at least four ANC visits (75.08%). Most also reported a preceding birth interval of 24 months or longer (88.99%) and had access to decision-making within their households (55.06%). Furthermore, over 62% of mothers expressed an active desire for more children, and nearly three-quarters (73.53%) visited a health facility in the past year **(Table 1).**

**Table 1**: Maternal and obstetrics- related factors in a study of spatial distribution and associated factors of low birth weight in Sub-Saharan Africa: based on DHS 2015-2024.

| Variables | Categories | Weighted frequency | Weighted  Percentage |
| --- | --- | --- | --- |
| Maternal age at first birth | Less/equal 18 year | 55,417 | 40.11 |
|  | Above 18 year | 82,747 | 58.89 |
| During pregnancy, given or bought iron tablets/syrup | Yes | 126,280 | 91.4 |
|  | No | 11,884 | 8.6 |
| Preceding birth interval | Less than 24 month | 15,213 | 11.01 |
|  | 24 and above | 122,951 | 88.99 |
| ANC visit during pregnancy | Not at all | 3,916 | 2.83 |
|  | 1_3 | 30,527 | 22.09 |
|  | Four and above | 103,721 | 75.08 |
| History of terminated pregnancy | Yes | 19,757 | 14.3 |
|  | No | 118,407 | 85.70 |
| Total children ever born | One | 27,619 | 19.94 |
|  | 2_3 | 56,314 | 40.65 |
|  | 4 and above | 54,588 | 39.41 |
| Last birth a cesarean section | Yes | 12,172 | 8.81 |
|  | No | 125,992 | 91.19 |
| Women access to decision making | Yes | 76,067 | 55.06 |
|  | No | 62,097 | 44.94 |
| Desire for more children | Active desire | 86118 | 62.33 |
|  | No active desire | 52046 | 37.67 |
| Visit health facility in the last 12 months | Yes | 101,596 | 73.53 |
|  | No | 36,568 | 26.47 |

1. **Child related factors**

The percentage of children was nearly equal by sex, with females at 50.07%. Most births were singletons (96.09%), and 38.21% were of second or third birth order **(Table 2).**

**Table 2:** child related factors in a study of spatial distribution and associated factors of low birth weight in Sub-Saharan Africa: based on DHS 2015-2024.

| Variables | Categories | Weighted frequency | Weighted  Percentage |
| --- | --- | --- | --- |
| Sex of child | Male | 68989 | 49.93 |
|  | Female | 69,175 | 50.07 |
| Twin birth | Yes | 5,408 | 3.91 |
|  | No | 132,756 | 96.09 |
| Birth order | 1 | 36,400 | 26.28 |
|  | 2_3 | 52,932 | 38.21 |
|  | 4 and above | 49,189 | 35.51 |

##

## Environmental related factors

The majority of participants (86.6%) lacked health insurance coverage, while two-thirds (66.6%) reported no difficulty accessing health facilities. Most mothers (72.6%) had media exposure and access to improved drinking water sources (74.34%), though nearly 80% relied on solid cooking fuels **(Table 3).**

Table 3: environmental - related factors in a study of spatial distribution and associated factors of low birth weight in Sub-Saharan Africa based on DHS 2015-2024

| Variables | Categories | Weighted frequency | Weighted  Percentage |
| --- | --- | --- | --- |
| Covered by health insurance | Yes | 18,520 | 13.40 |
|  | No | 119,644 | 86.60 |
| Distance from health facility | Big problem | 46195 | 33.44 |
|  | No problem | 91,969 | 66.56 |
| Media exposure | Yes | 100,292 | 72.59 |
|  | No | 37,872 | 27.41 |
| Source of drinking water | Improved | 102,712 | 74.34 |
|  | Unimproved | 35,452 | 25.66 |
| Cigarette smoke | Yes | 1,303 | 0.94 |
|  | No | 136,860 | 99.06 |
| Type of cooking fuel | Solid fuel | 110,401 | 79.91 |
|  | Clean fuel | 27,763 | 20.09 |

**Appendix 7:** Global spatial autocorrelation report for determining spatial clustering of low birth weight in SSA

**Spatial Autocorrelation Report**

| **Moran's Index:** | 0.234737 |  |
| --- | --- | --- |
| **z-score:** | 50.181145 | 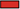 |
| **p-value:** | 0.000000 |  |


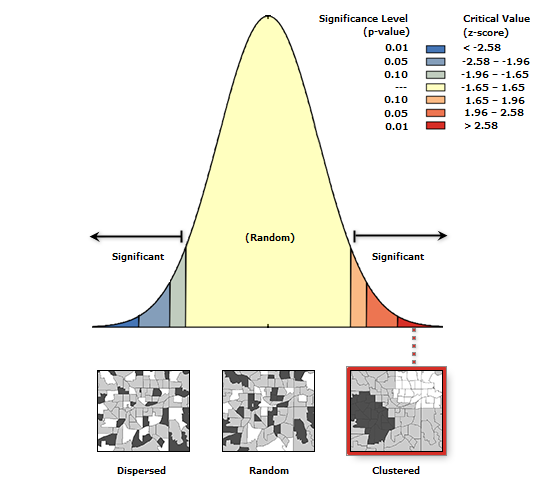


Given the z-score of 50.181145093, there is less than 1% likelihood that this clustered pattern could be the result of random chance.

Figure 1: spatial autocorrelation report for the study of spatial variation and determinants of low birth weight in SSA, DHS 2015-2024.

**Appendix 8:** Getis Ord General G report for determining high-low clustering of low birth weight in SSA

**High-Low Clustering Report**

| **Observed General G:** | 0.000641 |  |
| --- | --- | --- |
| **z-score:** | 45.427571 | 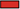 |
| **p-value:** | 0.000000 |  |


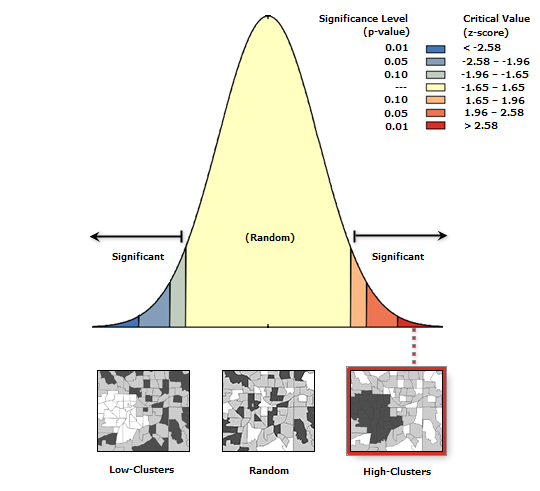


Given the z-score of 45.4275714425, there is less than 1% likelihood that this high-clustered pattern could be the result of random chance.

Figure 1: high low clustering report for the study of spatial variation and determinants of low birth weight in SSA, DHS 2015-2024

Appendix 9: Satscan analysis of low birth weight in SSA, DHS 2015-2024

| Cluster | Latitude | Longitude | Radius (Km) | Number of Enumeration area | LLR | P_VALUE | Relative Risk |
| --- | --- | --- | --- | --- | --- | --- | --- |
| Primary | -13.02149 | 33.466697 | 389.8665728 | 886 | 102.15 | P < 0.01 | 1.52 |
| Secondary | 14.180478 | -3.094051 | 428.5415966 | 179 | 80.96 | P < 0.01 | 2.35 |
| Secondary | 10.258134 | 39.530056 | 312.4356473 | 290 | 42.57 | P < 0.01 | 1.58 |
| Secondary | -20.616941 | 48.514331 | 592.7360911 | 559 | 37.51 | P < 0.01 | 1.29 |
| Secondary | 11.727902 | 9.387906 | 203.5289693 | 192 | 35.92 | P < 0.01 | 1.51 |
| Secondary | 15.490376 | -10.585827 | 198.5168031 | 299 | 25.41 | P < 0.01 | 1.48 |
| Secondary | 10.703894 | 15.116651 | 0 | 1 | 24.22 | P < 0.01 | 4.70 |
| Secondary | -1.895252 | 29.451324 | 92.40634988 | 386 | 20.06 | P < 0.01 | 1.42 |
| Secondary | 25.38257 | -11.597887 | 321.8025817 | 43 | 18.51 | P < 0.01 | 2.84 |
| Secondary | -12.271429 | 17.372913 | 0 | 1 | 17.98 | P < 0.01 | 4.47 |
| Secondary | 6.939825 | -10.887314 | 69.79563122 | 85 | 16.46 | P < 0.01 | 1.78 |
| Secondary | 8.4865039 | -0.80270401 | 30.71378386 | 5 | 15.31 | P < 0.01 | 3.68 |
| Secondary | 3.3433666 | 32.494876 | 0 | 1 | 14.01 | 0.018 | 6.73 |
| Secondary | 1.6075309 | 33.074226 | 0 | 1 | 13.84 | 0.021 | 7.31 |
| Secondary | -7.861994 | 13.114573 | 114.155374 | 52 | 13.84 | 0.021 | 1.67 |
| Secondary | 11.057105 | -0.096779 | 0 | 1 | 12.64 | 0.046 | 8.22 |
| Secondary | 4.629602 | 9.453911 | 0 | 1 | 12.60 | 0.046 | 6.17 |

**Appendix 10:** Exploratory regression

**Table 1:** Exploratory regression for identification of good candidate explanatory variables for the study of spatial distribution and its associated factor of low birth weight in SSA: DHS 2015-2024

| Variable | % Significant | % Negative | % Positive |
| --- | --- | --- | --- |
| Short birth interval | 100 | 0.00 | 100 |
| Age at first birth less than 18 month | 100 | 0.00 | 100 |
| Mom no job | 100 | 0.00 | 100 |
| No visit health facility in the last year | 100 | 0.00 | 100 |
| No insurance coverage | 100 | 0.00 | 100 |
| Twin birth | 100 | 0.00 | 100 |
| Women no education | 100 | 0.00 | 100 |
| No media exposure | 100 | 0.00 | 100 |
| Distance to health facility big problem | 97.14 | 0.00 | 100 |
| Women age 20-29 | 96.96 | 0.02 | 99.98 |
| Rural | 94.94 | 1.35 | 98.65 |
| Currently married | 94.86 | 0.93 | 99.07 |
| History of terminated pregnancy | 89.34 | 1.57 | 98.43 |
| Single | 85.58 | 8.32 | 91.68 |
| Last cesarean section delivery yes | 83.25 | 12.8 | 87.2 |
| No decision making power | 81.32 | 14.2 | 85.8 |
| Birth order | 80.1 | 15.2 | 84.8 |
| Soiled fuel | 75.34 | 12.96 | 87.04 |

Appendix 11: Diagnostic test for global regression models to determine factors associated with low birth weight in SSA: DHS 2015-2024.

| Regression Diagnostics | | | |
| --- | --- | --- | --- |
| Multicollinearity condition number | 4.269001 | | |
| Test on normality of error | | | |
| Test | DF | Value | Probability |
| Jarque bera test | 2 | 3.0063 | 0.22243 |
| Diagnostic for heteroskedaticity | | | |
| Test | DF | Value | Probability |
| Breush-Pagan test | 5 | 987.6342 | P <0.01 |
| Koenker Bassett test | 5 | 966.0277 | P <0.01 |
| Diagnostics for spatial dependence | | | |
| Test | MI/DF | Value | Probability |
| Moran’s I (error) | 0.2579 | 29.5885 | P <0.01 |
| Lagrange Multiplier (Lag) | 1 | 908.8629 | P <0.01 |
| Robust LM (Lag) | 1 | 137.7375 | P <0.01 |
| Lagrange Multiplier (Error) | 1 | 872.6121 | P <0.01 |
| Robust LM (Error) | 1 | 101.4867 | P <0.01 |
| Lagrange Multiplier (SARMA) | 1 | 1010.3496 | P <0.01 |

**Appendix 12:** global regression models

The spatial error model (SEM) emerged as the best-fitting global regression model, exhibiting the lowest AIC (52137.9) and the highest explanatory power (adjusted R² = 57%), outperforming both the OLS (R² = 50.5%) and spatial lag model (SLM) (R² = 55%) **(Table 1).** The spatial lag coefficient (0.2215, p < 0.01) indicates significant positive spatial dependence, suggesting that a 10% increase in low birth weight in neighboring districts is associated with a 2.2 percentage point rise in the focal district. Furthermore, the significant LAMBDA (λ) coefficient (0.208, p < 0.01) confirms spatial autocorrelation in the error terms, suggesting that unobserved factors affecting low birth weight are spatially clustered. Ignoring this spatial dependence would lead to biased and inefficient estimates, reinforcing the superiority of SEM over traditional OLS.

Key predictors retained their significance across all models but with varying magnitudes. Twin births (coefficient = 0.327 in OLS, 0.058–0.059 in spatial models) and lack of maternal healthcare visits (0.140 in OLS, 0.041–0.043 in spatial models) were strongly associated with low birth weight, while short birth interval, maternal unemployment, and limited media exposure also showed consistent effects **(Table 1).** Despite SEM’s superior performance, its assumption of spatial stationarity limits its ability to capture localized variations, necessitating further analysis using geographically weighted regression (GWR) and multiscale GWR (MGWR) to explore spatially heterogeneous relationships.

Table 1: Summary statistics and performance of global regression models for determining low birth weight in SSA; DHS 2015-2024

| Variables | Coefficient | | | St. error | | | Z-score | | | Pvalue | | |
| --- | --- | --- | --- | --- | --- | --- | --- | --- | --- | --- | --- | --- |
|  | OLS | SLM | SEM | OLS | SLM | SEM | OLS | SLM | SEM | OLS | SLM | SEM |
| Lag_low birth weight |  | 0.2215 |  |  | 0.009 |  |  | 25.141 |  |  | P<0.01 |  |
| Constant | 0.1645 | 0.313 | 0.442 | 0.010 | 0.006 | 0.004 | 16.435 | 52.074 | 96.92 | P<0.01 | P<0.01 | P<0.01 |
| Short birth interval | 0.086 | 0.028 | 0.028 | 0.002 | 0.001 | 0.001 | 37.783 | 29.340 | 28.23 | P<0.01 | P<0.01 | P<0.01 |
| No media exposure | 0.023 | 0.019 | 0.024 | 0.001 | 0.002 | 0.002 | 17.934 | 9.620 | 10.46 | P<0.01 | P<0.01 | P<0.01 |
| Donot visit health facility in last 1 year | 0.140 | 0.041 | 0.043 | 0.006 | 0.002 | 0.003 | 23.225 | 17.258 | 16.67 | P<0.01 | P<0.01 | P<0.01 |
| Twin birth | 0.327 | 0.058 | 0.059 | 0.014 | 0.003 | 0.003 | 24.183 | 16.758 | 17.51 | P<0.01 | P<0.01 | P<0.01 |
| Mom do not have job | 0.06 | 0.06 | 0.068 | 0.003 | 0.003 | 0.003 | 24.193 | 22.099 | 22.488 | P<0.01 | P<0.01 | P<0.01 |
| LAMBDA |  |  | 0.208 |  |  | 0.012 |  |  | 17.097 |  |  | P<0.01 |
| Performance of Global regression models | | | | | | | | | | | |  |
|  | **Ordinary Least Square (OLS)** | | | | **Spatial Lag Model (SLM)** | | | | **Spatial Error Model (SEM)** | | |  |
| $\mathbf{Adjusted R}^{\mathbf{2}}$ | 50.5 | | | | 55 | | | | 57 | | |  |
| AIC | 52437.8 | | | | 52275.2 | | | | 52137.9 | | |  |

Appendix 13: Performance of global and local regression models

Table 1: Performance of global and local regression models for estimating low birth weight in SSA DHS 2015-2024.

| Category | OLS | SLM | SEM | GWR | MGWR |
| --- | --- | --- | --- | --- | --- |
| $\mathbf{Adjusted R}^{\mathbf{2}}$ | 50.5 | 55 | 57 | 60 | 67.8 |
| AICc | 52437.8 | 52275.2 | 52137.9 | 50043.9 | 29092.9 |
